# Monetary policy effect on income and wealth inequality mechanism

**DOI:** 10.1371/journal.pone.0313304

**Published:** 2025-06-17

**Authors:** Zheng Wang, Yufei Chen, Wenjing Sun

**Affiliations:** 1 School of Medical Economics and Management, Anhui University of Chinese Medicine, Hefei, China; 2 Key Laboratory of Data Science & Innovative Development of Traditional Chinese Medicine, Philosophy and Social Sciences of Anhui Province, Hefei, China; 3 Uibe Business School, University of International Business and Economics, Beijing, China; University of Tunis El Manar Faculty of Economic Sciences and Management of Tunis: Universite de Tunis El Manar Faculte des Sciences Economiques et de Gestion de Tunis, TUNISIA

## Abstract

Since the 1990s, global income and wealth inequality has increased significantly, especially in developing countries, where the imbalance in wealth distribution has become increasingly prominent. This study seeks to thoroughly investigate the effects of expansionary monetary policy on income and wealth inequality, using China as a case study and employing extensive household survey microdata for empirical analysis. The findings indicate that expansionary monetary policy has significantly enhanced overall income and wealth levels. However, when considering the extent of wealth growth, it appears that affluent households have benefited more than their low- and middle-income counterparts, thereby widening the wealth gap. In addition, the real estate market boom played an amplifying role in this process, further deepening the impact of monetary policy on wealth inequality. The findings of this paper provide an important empirical basis for understanding the complex relationship between monetary policy and socio-economic inequality, and provide practical references for policymakers to consider the fairness of income and wealth distribution when formulating relevant monetary policies.

## Introduction

Since the 1990s, rising global income and wealth inequality has become an important topic of research in economics and sociology. According to the World Development Report (2022), this trend not only affects the sustainability of economic growth, but also poses serious challenges to social stability and equity. Income and wealth inequalities have triggered widespread concern about the effectiveness of economic policies and social redistribution mechanisms.

At the same time, the continued decline in long-term interest rates and the real neutral interest rate [1]. So that the traditional monetary policy regulation tools face new limitations. In this context, many countries have adopted loose monetary policy to stimulate economic growth. However, some studies have shown that loose monetary policy may exacerbate inequality, although the findings are somewhat controversial [2,3]. The contradictory nature of these studies and the lack of systematic comparative analysis make it possible for monetary authorities to ignore their far-reaching effects on income and wealth distribution when formulating policies.

The real estate market plays a key role in the distribution of wealth, with data showing that real estate accounts for 75.7% of the net wealth of Chinese households, making fluctuations in house prices have a profound impact on the distribution of household wealth. Drawing on Čermáková et al. [4] (2023), the relationship between transaction volumes and house prices is key to understanding real estate market dynamics. Loose monetary policy typically leads to higher real estate prices, allowing greater wealth appreciation for households that own real estate, while households that fail to invest in real estate are at risk of relative poverty. This phenomenon is particularly evident in the widening urban-rural and regional gaps. Studies have shown that uneven development of real estate markets exacerbates wealth inequality [5,6], therefore, policy makers need to focus on the interaction between transaction volumes and house prices in order to develop more effective policies to mitigate inequality.

In today’s global economic environment, differences in housing affordability are becoming increasingly significant, especially among different age groups. Younger generations, as one of the most vulnerable groups, are facing unprecedented challenges in homeownership. According to Čermáková et al. [4], changes in real estate prices are closely related to the number of transactions in the market, with increased volumes usually signaling higher prices, but prices tend to show a downward rigidity trend, which further reduces the ability of young homebuyers to purchase a home in the face of the dual pressures of high prices and limited income [1]. In addition, the implementation of macroprudential policies has already significantly limited the ability of young homebuyers to finance their homes through the introduction of metrics such as the debt-to-income (DTI) and debt service-to-income (DSTI) ratios. These policies, while aimed at stabilizing financial markets, have inadvertently made it more difficult for young people to enter the housing market [7]. Studies have shown that the younger generation has experienced the least change in purchasing their own homes, not only due to the continued rise in house prices, but also closely related to lagging income growth and other socio-economic factors [8].

While low interest rates and expansionary monetary policies have stimulated the real estate market in the short term, their long-term effects tend to exacerbate wealth inequality, making young people face higher barriers to entry when purchasing a home. In addition, young people often lack sufficient savings and credit history, putting them at a disadvantage when it comes to obtaining a mortgage [9]. This phenomenon manifests itself differently across countries and regions; for example, in some European countries, government policies to support first-time buyers have failed to adequately alleviate the housing burden on young people [10]. Despite the decline in overall poverty levels in some countries, housing problems remain prominent. Łuczak and Kalinowski [11] point out that many young people, even in relatively improved economic environments, continue to be squeezed by a combination of high house prices and low incomes, resulting in their ability to purchase a home being limited. This phenomenon is particularly evident in Central and Eastern European countries, where young people’s housing affordability is often disproportionate to local economic development, becoming an important barrier to their economic independence and social mobility. As a result, the younger generation is not only confronted with high housing prices, but also with the effects of inequality brought about by policy and market changes.

The analysis of economic factors and supply and demand is also crucial in the study of the housing market. When considering a property, homebuyers must comprehensively assess the different costs of new versus older units, not only in terms of purchase price, but also in terms of long-term operation and maintenance costs. This comprehensive perspective provides a more accurate framework for understanding the economic characteristics of different types of housing. Life cycle cost analysis (LCC) serves as an effective assessment tool that can help homebuyers make more rational choices when considering new construction versus historic buildings. According to Pojar et al. [12], LCC analysis plays an important role in guiding the maintenance and rehabilitation of cultural heritage buildings. By considering the entire life cycle of a building, including the design, construction, operation, and eventual demolition or renovation phases, this analysis can provide investors with a long-term assessment of economic benefits.

In their study, Hromada et al. [13] emphasized that it is a common misconception to select a building solution based solely on the lowest acquisition cost. In fact, many homebuyers may overlook the long-term impact of operation and maintenance costs, which can lead to subsequent financial burdens.The use of LCC tools can not only help evaluate the economics of different design variants, but also identify the components or equipment that have the greatest impact on the total life-cycle costs, thus providing a more holistic economic perspective for homebuyers. When analyzing different types of buildings, homebuyers should recognize the differences in cost structures between new construction and historic buildings. New buildings typically have higher energy efficiency and modernization features in their design and material selection, but their initial investment may be relatively high [14]. In contrast, historic buildings can be more expensive to maintain, especially if their cultural and historical value is ensured, however, appropriate restoration and renovation can also increase their market value [15].

This study quantitatively analyzes the impact of expansionary monetary policy on income and wealth inequality using data from the China Family Panel Study (CFPS). Preliminary results show that expansionary monetary policy not only significantly increased overall income and wealth levels, but also that the growth in wealth significantly outpaced the growth in income. Specifically, the richest 10 per cent of households benefited 6.2 times more than the least wealthy 20 per cent, suggesting an increase in wealth concentration. In addition, urban households benefited from the policy to a significantly greater extent than rural households, especially those with higher levels of financial literacy, whose wealth grew more rapidly. This phenomenon reflects the unevenness of expansionary monetary policy in promoting wealth accumulation, especially when driven by the real estate market, which further exacerbates socioeconomic inequality. This study will reveal how expansionary monetary policy contributes to the concentration of wealth in a small number of households, thereby exacerbating overall social inequality. It has been shown in the literature that wealth inequality changes more significantly under the influence of monetary policy [16–18]. This study will fill the gap of previous studies that mainly focus on income inequality and further reveal the mediating role of the real estate market in the impact of expansionary monetary policy on wealth inequality.

## Literature review and theory analysis

This study will comprehensively review the existing literature and deeply analyzne the relationship between expansionary monetary policy and economic inequality. By systematically sorting out relevant studies, it will reveal the views and findings of different scholars in this area in order to lay a solid theoretical foundation for this study. In addition, the study will provide a theoretical framework to help understand the dynamic mechanisms of income and wealth disparities, especially the responses and adjustments in the context of policy changes.

Based on the literature analysis, the study will develop key hypotheses that will form the core foundation of our research. Through the establishment of these hypotheses, the impact of expansionary monetary policy on different households will be explored more effectively, and in turn, its specific role on income and wealth inequality will be analyzed.

### Assumption 1 about household income components

Households’ sources of income are mainly categorized into labor income (LE) and investment income (II), both of which play an important role in income inequality analysis. Labor income is one of the main sources of household income and includes wages, salaries, income from agricultural or business operations, and other employment-related sources of income. According to economics, labor income is usually the focus of income inequality assessment because it directly reflects an individual’s ability to work and the market demand. Hansen & Prescott [19] point out that the stability of labor income is particularly important during economic fluctuations, especially during recessions, when labor income can be greatly affected, leading to a deterioration of a household’s economic situation. In addition, the distribution of labor income is affected by a variety of factors, including education level, industry choice, and regional differences [20]. These factors lead to significant inequality in labor income among different groups, thus further exacerbating overall income inequality.

In contrast to labor income, investment income comes mainly from gains on financial assets and real estate, including capital or property income. During periods of economic growth, investment income usually increases substantially and contributes more to the income growth of the rich [21]. This phenomenon suggests that income earned by the rich through investment tends to grow faster than the wages of ordinary workers, thus increasing income inequality. It has also been found that the growth of investment income tends to follow a pro-cyclical pattern, i.e., investment returns rise sharply during booms, while remaining relatively stable or declining during recessions [22]. This contrasts with the countercyclical behavior of labor income, which tends to be hit harder in recessions, leading to greater instability in household income.

In the United States, rising property income inequality has been identified as one of the main factors affecting income inequality [23]. With the development of the capital market, the wealth of wealthy households mainly comes from investment returns, and the distribution of these returns tends to be more inclined to exacerbate income disparities in society. In China, although property income accounts for a relatively small proportion of total income, its degree of inequality exceeds that of labor income [24]. This suggests that the degree of concentration of wealth can lead to severe income inequality despite the relatively low overall economic level. This is particularly evident in the context of the rapid development of the real estate market, where the gains from asset appreciation flow mainly to a small number of affluent groups, further exacerbating social stratification.

Consequently, household income (*I*) can be expressed as the sum of labor earnings (*LE*) and investment income (*II*):


I=LE+II

where labor earnings (*LE*) are influenced by employment (*E*), a function shaped by expansionary monetary policy (*MP*) and various other economic factors:


LE=f(E,MP,...)

Investment income (*II*) is influenced by the returns on financial assets and real estate (*R*), together with the structure of the portfolio (*P*). It is important to highlight that an expansionary monetary policy can impact the returns on financial assets:


II=f(R,P,MP,...)

### Assumption 2 about household wealth composition

The definition of household wealth consists of the stock of wealth plus the net value of new income minus expenditures, a composition that clearly reflects the financial health of the household. Household wealth is not only a reflection of the family’s economic strength, but also an important guarantee of the family’s future quality of life [25]. The source of property income is usually derived from a household’s stock wealth, which suggests a strong link between income and wealth [26] (Piketty, 2014). However, despite the positive correlation between household income and wealth, empirical data suggests that the correlation between the two is relatively weak. For example, the 2012 and 2014 Chinese Family Panel Studies (CFPS) indicate that the correlation coefficients between household income and property income are 0.37 and 0.26, respectively. In contrast, the 2013 Survey of Consumer Finances (SCF) in the U.S. shows a correlation coefficient between income and net wealth of 0.33.This result underscores the fact that income is not the sole influence on the wealth accumulation factor.

There are a number of factors that affect wealth accumulation, including consumption patterns, savings habits and investment returns. A household’s consumption pattern directly affects the amount of money it has available for savings and investment, which in turn affects the long-term growth of wealth [27]. Overconsumption may lead to insufficient savings, thus hindering the accumulation of wealth. In addition, good saving habits can help households maintain financial stability during economic fluctuations, while the level of investment returns can have a significant impact on wealth appreciation. High-return investments can accelerate wealth growth, while low-return investments can lead to stagnant or shrinking wealth [28].

Consequently, household wealth (*W*) can be expressed as the sum of real estate wealth (*RE*) and financial asset wealth (*FA*):


W=RE+FA

Where real estate wealth (*RE*) is influenced by property values (*V*), which, in turn, can be influenced by loose monetary policy:


RE=f(V,MP,...)

Financial asset wealth (*FA*) depends on the composition of the investment portfolio (*P*) and the returns on financial assets (*R*). Loose monetary policy can indeed affect financial asset returns:


FA=f(P,R,MP,...)

### Assumption 3 about expansionary monetary policy characteristics

Monetary policy encompasses the various strategies adopted by the monetary authority to regulate the money supply and monitor interest rates, especially short-term interest rates. This includes adjusting the reserve ratio and base money to effectively control inflation and promote economic growth. Expansionary monetary policy (MP) specifically focuses on lowering interest rates and increasing the money supply (M2) to promote economic activity. However, although monetary policy across countries does not directly target income or wealth inequality, empirical studies have been conducted to show that monetary policy may lead to redistributive consequences [29]. Central bankers have long been aware of the distributional effects of monetary policy, with Greenspan [30] noting that the distributional effects are particularly pronounced when policy strength is atypical. This effect becomes particularly pronounced when policy strength is atypical. This is closely related to the different characteristics of households. Differences in household sources of income, asset holdings, financial literacy, saving behavior and access to financial services generate different pathways through which monetary policy can affect inequality [31].

The overall impact of inequality remains controversial. Bernanke [32] noted that it is unclear whether the overall impact of monetary policy increases or decreases inequality, which requires further research on the transmission channels of income distribution and their distributional effects. The channels of impact of expansionary monetary policy mainly include the income heterogeneity channel, which increases employment opportunities by reducing unemployment and promoting economic expansion [33]. However, there are significant differences in the impact of expansionary monetary policy on unemployment and income across demographic groups [34]. The incomes of high-income households are mainly affected by changes in hourly wages, while low-income households are more affected by the number of hours worked.

In addition, the impact of savings redistribution is another important issue [35]. When interest rates fall, savers have less interest income, while borrowers benefit from lower interest payments. In general, low-income households tend to save less, while the middle class and middle-aged tend to carry more long-term nominal debt. In contrast, older and wealthier households are typically not net savers and are therefore more affected by falling interest rates [36]. Changes in inflation likewise affect household welfare differently, with lower-income households more adversely affected by inflation because they hold a higher proportion of cash. Inflation not only reduces the purchasing power of cash, but also exacerbates inequality as a regressive consumption tax [36,37]. Finally, studies on the portfolio channel have shown that monetary policy has a significant impact on households’ financial assets and investment returns, especially interest income [38].

The structure of these portfolios varies from household to household, leading to varying impacts of expansionary monetary policies on wealth redistribution. In general, such policies tend to increase asset prices, providing an advantage to households with substantial real estate and financial assets. Higher stock prices also improve corporate balance sheets and profit outcomes, benefiting higher-income households [39–41].

### Assumption 4 about sensitivity of financial asset returns

In exploring the sensitivity of returns on financial assets, we assume that returns on financial assets (R) are more susceptible to expansionary monetary policy than labor income (LE). Specifically, adjustments in monetary policy directly affect asset prices, which in turn affects households’ financial incomes. This impact mechanism can be understood through the volatility of asset prices. Expansionary monetary policy usually leads to lower interest rates, which in turn increases the prices of financial assets such as stocks, corporate bonds and government bonds [42]. When interest rates are lowered, the cost of borrowing decreases and the willingness of firms and consumers to raise funds increases, leading to more investment and consumption activities [43]. This economic stimulus will not only increase the profitability expectations of firms, but will also increase investor confidence in future economic growth, thus driving up the prices of financial assets.

In addition, loose monetary policy affects investors’ risk appetite [44]. In a low-interest rate environment, returns on fixed-income assets decline, and investors tend to seek high-yield investment opportunities, such as stocks and real estate. This shift not only increases the demand for these assets but also pushes up their prices further [45]. As the prices of financial assets rise, the household wealth effect increases significantly, and consumption expenditure then increases, thus creating a virtuous cycle. This mechanism suggests that monetary policy affects asset prices not only by directly adjusting interest rates, but also indirectly by changing investor behavior and market expectations on the returns of financial assets.

It is worth noting that wealthy households typically hold more financial assets, especially stocks and bonds, and thus monetary policy easing measures may substantially increase the capital income of these households and thus exacerbate wealth inequality [46,47]. In contrast, labor income (LE) has a more fixed pay structure and its short-term fluctuations are relatively limited. This implies that adjustments in labor income may not be as important as returns to financial assets in the early stages of monetary policy implementation. This difference contributes to the importance of wealth transfers, as asset price adjustments triggered by loose monetary policy can lead to a rapid increase in the wealth of wealthy households and a relatively slow increase in the wealth of low- and middle-income households, further widening the gap between the rich and the poor.

The mechanisms by which loose monetary policy affects household income and wealth dynamics emphasize key differences in its impact on wealth. Specifically, returns on financial assets are more sensitive to changes in monetary policy than are labor incomes, suggesting that the implementation of the policy not only affects the level of household incomes, but may also exacerbate pre-existing inequalities in the wealth accumulation mechanism. Hypothesis 4 therefore argues that the higher sensitivity of returns on financial assets than labor income reflects the far-reaching impact of expansionary monetary policy on the distribution of household wealth.

By analyzing more deeply how monetary policy affects the return on financial assets, this study is able to understand more fully the role of monetary policy in wealth inequality. The sensitivity of the rate of return on financial assets and its comparison with labor income reveals the complexity of monetary policy and its far-reaching impact on the distribution of wealth in modern economies.

## Empirical strategy

### Model specification

In order to examine the impact of expansionary monetary policy on household wealth and income, we establish a benchmark model with household per capita income and per capita wealth as dependent variables, and monetary policy as an independent variable based on the literature discussed above. The model specification is as follows:


$$INC_{i,t}=\beta_{0}+\beta_{1}MP_{t}+\beta_{cv}CV_{i,t}+NF_{i}+YF_{t}+T+\xi_{i,t}$$ (1)

$$NetWorth_{i,t}=\beta_{0}+\beta_{1}MP_{t}+\beta_{cv}CV_{i,t}+NF_{i}+YF_{t}+T+\xi_{i,t}$$ (2)

where *i* = 1, 2, … denotes the household identity; *t* = 1, 2, … denotes the period; $NF_{i}$ represents the fixed effect of households, which accounts for an unobserved factor related to specific households and a disturbance term of individual heterogeneity; $YF_{t}$ indicates the fixed effect of the year, which is the change of macroeconomic situation faced by households in different years, and is a disturbance term of time heterogeneity; $\xi_{i,t}$ represents the error term; and *T* is the time trend term. The explained variables $INC_{i,t}$ and $NetWorth_{i,t}$ represent the per capita income and per capita net wealth of household *i* in period *t*. Explanatory variables $MP_{t}$ represent expansionary monetarypolicy in period *t*. Control variables $CV_{i,t}$ include household socio-economic characteristics such as household net property $W\_Total_{i,t}$, household net income $H\_Income_{i,t}$, average education level of the household labor force $Edu\_LF_{i,t}$, the proportion of household labor force $P\_LF_{i,t}$, household size $familysize_{i,t}$, and household urban and rural attributes $UR\_Attr_{i,t}$. We do not include time-invariant variables such as gender and political status in the model specification because we control the fixed effects at the household level.

This study uses money supply (M2) as the main macro variable to explore its impact on household income and wealth. We are inspired by changes in economic fundamentals and central bank policies to construct an endogenous monetary policy rule, drawing on the Taylor-like rule proposed by Chen et al. [48], and taking into account changes in inflation and GDP growth rates in the context of China. For the choice of independent variables, money supply (M2) is used as a macro policy indicator to reflect the degree of monetary policy easing or tightening, which directly affects households’ consumption and investment capacity. The dependent variables, on the other hand, include household income and household wealth, with the former covering wages, investment income, etc. and the latter involving real estate, savings and other assets, reflecting the level of household wealth accumulation.

To alleviate the endogeneity problem caused by two-way causality, this study will also introduce control variables, including inflation rate and GDP growth rate, which affect the real purchasing power of households and the overall growth of the economy, thus indirectly affecting household income and wealth. For the model, we use the Ordinary Least Squares (OLS) method to estimate the baseline model in the form of a household income or wealth equal to the constant term plus the weighted sum of the money supply and control variables, plus an error term. In order to gain a deeper understanding of the impact of expansionary monetary policy on different income groups, we categorize households into low-income, middle-income, and high-income groups and conduct separate regression analyses for each group, with the classification criteria being income per capita and net wealth per capita, in order to assess the impact of the policy on households in different economic situations.

Through the above model, we will be able to understand the changes in the impact of expansionary monetary policy on different income groups and assess its potential impact on income inequality, and this analysis will provide an important reference for policy makers. To ensure the reliability of the findings, we will also conduct robustness tests, including the use of alternative samples and different regression methods, as well as the introduction of other proxy variables to verify the robustness of the independent factors.

### Definitions of variables

In this study, the selection and definition of variables are crucial in analyzing the relationship between household income and wealth. The relationship between household income and wealth is significant. The dependent variable is per capita household income, which serves as a key economic indicator that accurately represents the financial status of households and aligns with findings from earlier research, thereby improving the comparability of outcomes. Per capita household income is determined by dividing the total household income by the number of individuals in the household and encompasses a variety of income sources, such as wages, business earnings, transfer payments, property income, and other forms of income.

In order to gain a deeper understanding of the impact of each type of income on a household’s economic situation, household income is further subdivided into several categories: wage income (including after-tax wages, bonuses, and in-kind benefits), business income (covering net income from agriculture and self-employment), transfer income (e.g., pensions, subsidies, and social donations, etc.), property income (income from renting land, houses, and means of production), and other income (e.g., family and friends support and donations, etc.). This categorization not only helps to present a clear picture of the income structure, but also provides an important basis for subsequent analysis.

In examining wealth, household wealth per capita was chosen as the explanatory variable to analyze the connection between wealth accumulation and income. The household wealth was categorized into five groups: land, real estate, financial assets, productive fixed assets, and consumer durables. The valuation method was based on McKinney’s (1993) approach, which posits that 25% of a household’s total agricultural income is derived from land. The real estate segment encompasses the value of existing homes and other properties, while financial assets cover liquid investments such as deposits, stocks, and bonds. Productive fixed assets include agricultural equipment, whereas consumer durables consist of common household items like automobiles and televisions.

M2, as a proxy variable for monetary policy, is included in the model in its annual logarithmic form to avoid the problem of heteroskedasticity. Considering the many changes in the statistical caliber of M2 experienced, this study uses macroeconomic time series data from Higgins and Zha to ensure data consistency and comparability. This paper chooses household wealth per capita as the explanatory variable, which is consistent with the studies of Xie et al. and Wan et al. [6,49]. Through the definition and classification of the above variables, this study seeks to comprehensively and systematically analyze the dynamic relationship between household income and wealth (Table 1).

**Table 1:** Definition, description and descriptive statistics of variables.

Variable	Variable description	Calculation method	Mean value	Standard deviation	Min. value	Max. value
INC	Average distribution of income per family member	Ratio of net household income to household size, then taking the natural logarithm	7.203	1.197	3.784	13.756
NetWorth	Average net wealth per household member	Net household wealth divided by the natural logarithm of the number of household members	11.675	1.292	−1.272	19.082
M2	Monetary policy	M2 balance of annual money supply. Use the natural logarithm.	5.426	0.166	5.567	4.914
P_LF	Proportion of household members participating in the labor market	Proportion of household working population aged 16–60 and not in school to total population	0.545	0.273	0	1
Edu	Average level of education of persons working in the labor market	Using dummy variables to indicate the level of education: 1 for illiterate and semi-illiterate, 2 for elementary school and below, 3 for lower secondary school, 4 for upper secondary school (middle school), 5 for specialized school, 6 for bachelor’s degree, 7 for master’s degree and 8 for doctorate.	2.170	0.850	1	7
FamSize	Family size	Number of persons with economic resources in the household	3.066	1.778	1	9
H_Value	Total value of housing owned by households	Sum of all housing values in natural logarithms	11.347	1.374	9.805	16.776
W_Total	Net household wealth	Total net household assets = household cash and deposits + real estate value + operating asset value + financial asset value-household liabilities. Use the natural logarithm during regression.	470237.8	894131.6	−40660	4764420
HW_Total	Total household wealth	Total household assets = household cash and deposits + real estate value + operating asset value + financial asset value. Use the natural logarithm during regression.	384343.1	596203.1	2750	6192640
H_Income	Household income	Household income = wage income + operating income + property income + transfer income + other income. Use the natural logarithm during regression.	43098.7	41809.5	535	228960
FinLit	Financial literacy	1 indicates a level of financial literacy greater than or equal to that of peers, 0 indicates less than that of peers.	0.149	0.287	0	1
UR_Attr	Attributes and characteristics of the family in an urban or rural environment	1 for urban households, 0 for rural households.	0.331	0.600	0	1
H_PM	Whether there are party members in the family	1 for yes and 0 for no.	0.145	0.421	0	1
Sample_Prov	Sample province identification	1-5 for 5 major provinces and 6 for other provinces.	5.855	1.763	1	6
NS	National sub-sample or not	1 for yes and 0 for no.	0.706	0.481	0	1

### Data processing

Based on the China Family Panel Study (CFPS) database, this study aims to provide an in-depth analysis of the dynamics of economic and non-economic welfare characteristics of Chinese households. During data processing, systematic and rigorous steps were taken to ensure the accuracy and reliability of the data used, thus providing a solid foundation for subsequent analysis. This study utilizes micro-level data from the China Family Panel Studies (CFPS) spanning five issues between 2010 and 2020. The data was from the Supporting Information (S1 Data).

During the data cleaning phase, the raw data were thoroughly reviewed and screened. For the treatment of missing values, all records with missing data were excluded to maintain the integrity of the overall data. At the same time, records of households with an income share not equal to 1 were excluded to ensure logical consistency and validity of the data. In addition, records with per capita household income (INC) less than 1 were excluded to avoid extreme values adversely affecting the analysis results. To further enhance data quality, only records with credibility and cooperation level of 1 were retained to ensure the completeness and reliability of the sample.

In terms of outlier treatment, the bilateral 1% Winsorization method was adopted to effectively treat continuous variables such as per capita household income (INC) and average net wealth per household member (NetWorth). This method helps to replace extreme values, thus reducing the interference of outliers in the results. Meanwhile, in order to satisfy the basic assumptions of statistical analysis, the natural logarithmic transformation was applied to the heteroskedasticity variables to make their data distribution closer to the normal distribution, which enhances the robustness and explanatory power of the subsequent regression model.

Table 2A showed the income sources as a Share of Household Income (2018). Table 2B showed the wealth sources as a Share of Household Wealth (2018).This sample is not only highly representative, but also able to effectively support the subsequent empirical analysis. Notably, data from five consecutive survey versions were analyzed to ensure data comparability, and all income and wealth data were based on CFPS 2010 baseline comparable variables. This series of rigorous data processing steps provides solid and reliable data support to study changes in the economic and non-economic welfare characteristics of households and ensures the scientific validity and effectiveness of the findings.

**Table 2:** Income sources and wealth sources as a share of household income and wealth (2018).

Income quintiles						
Income source	1st	2^nd^	3rd	4th	5^th^	total
**A:** Income Sources as a Share of Household Income (2018).						
Wage	17.5	48.9	66.6	73.2	63.4	57.9%
Business	19.8	16.2	12.6	7.1	3.3	9.7%
Property	3.9	2.6	1.5	1.4	2.6	2.3%
Transfer	42.1	20.3	13.1	14.8	26.9	6.7%
Other	15.1	11.4	6	3.5	3.7	23.1%

**Wealth quintiles**						
**Wealth source**	**1st**	**2nd**	**3rd**	**4th**	**5th**	**total**
**B: **Wealth Sources as a Share of Household Wealth (2018).						
Land	17.8	13	8.5	4.9	0.7	3.0%
Real estate	63.5	67.1	70.8	73.5	77.8	75.7%
Financial asset	9.9	11.4	11.3	11.9	10.9	11.1%
Fixed	2.2	1.8	2	2.2	6.3	5.0%
Durable	6.6	6.7	7.3	7.5	4.3	5.3%

Data source: CFPS 2018 survey.

### Descriptive statistics

The study focuses on the impact of monetary policy on household income and wealth. Table 1 shows the regression results under different variables revealing the multidimensional characterization of household economic status.

In terms of the impact of money supply (M2), its positive effect on household income (H_Income) and wealth (W_Total) is significant. Specifically, the regression coefficient of M2 on H_Income is 1.646*** and on W_Total is 2.141***. This suggests that the monetary policy expansion implemented by the People’s Bank of China after the financial crisis in 2008 directly contributed to the improvement of H_Income and W_Total. During the period from 2009 to 2017, the average annual growth rate of M2 reached 14.9%, which was significantly higher than the growth rate of GDP and the growth rate of the CPI, reflecting a significant improvement in the economic situation of households against the backdrop of the loosening of monetary policy.

The impact of education level (Edu_LF) is also noteworthy. Although the regression coefficient of 0.124 for Edu_LF on H_Income does not reach the significant level, its effect on W_Total is significant (0.070**), indicating the importance of education in the accumulation of household wealth. In the process of rapid urbanization and economic transformation in China, increased educational attainment provides more economic opportunities for families and strengthens their economic capacity.

Labor force participation (P_LF) also exhibits a significant positive effect in the analysis, with a regression coefficient of 0.585*** for H_Income and 0.224** for W_Total. This result reflects the critical role of P_LF on the economic status of households during the period of economic restructuring in China. With the acceleration of urbanization, more and more rural laborers enter the cities, enhancing the economic vitality of households.

In terms of family size (FamSize), the data reveal its negative impact on H_Income and W_Total, with a coefficient of −0.027* for H_Income and −0.209*** for W_Total. This phenomenon may reflect the dilution of resource allocation as a result of the increase in FamSize, especially in the context of the urbanization process, where changes in the structure of the household may affect the economic performance of the household.

In addition, the regression coefficient for total income (H_Income) is 0.111***, emphasizing the important role of income level in the accumulation of household wealth (W_Total). Accompanied by economic growth and consumption upgrading, the accumulation of W_Total shows a positive feedback relationship with the increase in H_Income. The results of the regression analysis in Table 1, with sample sizes of 24338, 16455, 18257, and 20092, respectively, controlling for individual and time effects, validate the far-reaching impact of monetary policy adjustments on H_Income and W_Total.

## Results

### Baseline regression results

T This section employs a panel regression model utilizing micro-level data for the analysis, where the individual sample size exceeds the time period [50]. This approach ensures consistent estimations while avoiding concerns related to pseudo-regression. To reduce the risk of pseudo-regression, variables reflecting the relationship between M2 and average distribution of income per family member (INC) are incorporated into the model. For model selection, the Hausman test favors the use of a fixed effects model, and the time dummy variable test does not dismiss the hypothesis of time fixed effects. Additionally, robust standard errors were applied to address potential heteroskedasticity. The regression outcomes are presented in Table 3.

**Table 3:** Regression results of monetary policy on income and wealth.

	income	income	wealth	wealth
M2	1.245***	1.372***	2.506***	2.344***
	(0.036)	(0.051)	(0.049)	(0.057)
Edu		0.131***		0.034*
		(0.021)		(0.021)
P_LF		0.550***		0.159**
		(0.050)		(0.059)
W_Total		0.105***		
		(0.007)		
FamSize		−0.028***		−0.150***
		(0.010)		(0.010)
H_Income				0.128***
				(0.006)
Cons.	0.566***	−1.165***	−2.018***	−0.986***
	(0.156)	(0.189)	(0.198)	(0.270)
Individual effect	Yes	Yes	Yes	Yes
Time effect	Yes	Yes	Yes	Yes
Time trend	Yes	Yes	Yes	Yes
N	34055	29835	30424	25839

Note: Clustering robust standard error in parentheses. ***, ** and * indicate significance at 1%, 5% and 10% levels, respectively. N is the number of samples.

According to the regression analysis, the coefficients of M2 are significant in all models at 1.245 (INC) and 2.506 (NetWorth), both at the 1% significance level, indicating that an increase in money supply has a positive impact on the growth of household income and wealth. The effect of education level (Edu) on household income and wealth is equally significant. The coefficient of education is 0.131 (1% significance) in the INC model and 0.034 (10% significance) in the NetWorth model, showing the close relationship between education and increased household income.

In addition, the proportion of household members participating in the labor market (P_LF) shows a significant positive impact in both the INC and NetWorth models with coefficients of 0.550 (1% significance) and 0.159 (5% significance), respectively, highlighting the key role of the labor market on the economic status of households. Total household wealth (HW_Total) also shows a significant positive impact in the NetWorth model with a coefficient of 0.105 (1% significance), further supporting the relationship between wealth accumulation and household resources. The negative impact of family size (FamSize), on the other hand, is −0.028 (1% significance) in the INC model and −0.150 (1% significance) in the NetWorth model, revealing the potentially unfavorable impact of household size on economic status.

After testing for differences between groups using the SUEST methodology, the regression results in Table 4 reveal a significant effect of income and wealth on each group. Specifically, in the bottom 50% income group, the coefficient of M2 is −0.657, indicating a significant negative impact of money supply on this group. In contrast, in the middle 40% and top 10% income groups, the coefficient of M2 is 0.310 and 0.832, respectively, showing a positive impact of money supply on both groups, indicating that the policy effects differ significantly across income levels.

**Table 4:** Regression results in different groups.

	Bottom 50%		Middle 40%	Top 10%	Bottom 50%		Middle 40%	Top 10%
	Bottom 20%	Next 30%			Bottom 20%	Next 30%		
	income	income	income	Income	wealth	wealth	wealth	wealth
M2	−0.657***	0.310***	0.832***	0.681***	0.305*	0.403***	0.928***	2.055***
	(0.154)	(0.032)	(0.027)	(0.061)	(0.121)	(0.037)	(0.041)	(0.118)
Edu	0.074	0.034**	0.041***	0.035	0.090	−0.021	0.028*	0.057
	(0.057)	(0.013)	(0.009)	(0.024)	(0.048)	(0.012)	(0.014)	(0.031)
P_LF	0.096	0.036	0.143***	0.045	−0.065	0.056	0.073*	0.032
	(0.148)	(0.039)	(0.029)	(0.061)	(0.143)	(0.032)	(0.035)	(0.079)
W_Total	0.043*	0.028***	0.042***	0.047***				
	(0.022)	(0.007)	(0.006)	(0.014)				
FamSize	0.035	−0.003	−0.035***	−0.072***	−0.053*	−0.050***	−0.109***	−0.095***
	(0.024)	(0.005)	(0.004)	(0.015)	(0.023)	(0.008)	(0.006)	(0.025)
H_Income					0.067*	0.028***	0.030***	0.019
					(0.022)	(0.005)	(0.006)	(0.024)
Cons.	10.596***	6.657	4.780	6.914	6.606***	7.797***	5.801***	3.198***
	(0.716)	(0.172)	(0.149)	(0.395)	(0.605)	(0.194)	(0.174)	(0.469)
Individual effect	Yes	Yes	Yes	Yes	Yes	Yes	Yes	Yes
Time effect	Yes	Yes	Yes	Yes	Yes	Yes	Yes	Yes
Time trend	Yes	Yes	Yes	Yes	Yes	Yes	Yes	Yes
N	4389	7176	11929	2630	5031	7607	9749	2330

Note: Clustering robust standard error in parentheses. ***, ** and * indicate significance at 1%, 5% and 10% levels, respectively. N is the number of samples.

The education variable (Edu) shows a significant positive impact in the middle 40% and top 10% groups with coefficients of 0.034 and 0.041, respectively, indicating that education has a positive contribution to economic activity in the middle and upper income groups. However, the insignificant impact of the variable in the bottom 50% group may reflect the more pressing economic challenges faced by the low-income group.

The coefficient of the proportion of household members participating in the labor market (P_LF) also shows differences across groups, with a coefficient of 0.143 for the top 10% group, indicating that labor force participation has a significant enhancing effect on the economic status of the high-income group. In contrast, the bottom income group does not show significance, suggesting that the labor force participation rate of the low-income group has a limited impact on their economic status. Family size (FamSize) showed significant negative coefficients of −0.035 and −0.072 in the bottom 20% and middle 40% groups, respectively, pointing out that family size may adversely affect the economic status of low-income and middle-income families.

The research indicates that the average educational attainment (Edu) and the share of the labor force within a household do not significantly influence the income growth of high-income families. Nevertheless, total household wealth (HW_Total) remains an important factor, highlighting the significant role of property in their financial resources. Additionally, regarding wealth accumulation, average distribution of income per family member (INC) has a less pronounced effect on top-tier households, implying that their wealth is primarily influenced by asset appreciation rather than by increased income streams. This conclusion aligns with the results of Li et al. (2021) and Guo and Tao (2022).

### Robustness test

This section aims to verify the consistency and reliability of the impact of expansionary monetary policy on household income and wealth through robustness tests. The differential effects of the policy across income and wealth levels are further explored by analyzing different samples to ensure the robustness of the findings.

Table 5 presents the outcomes of robustness tests conducted on various aspects of household income and wealth in relation to expansionary monetary policy. The data reveals that M2 has a notably negative effect on the average distribution of income per family member (INC) of the lower 50% of households, with a coefficient of −0.715 (significant at the 1% level). Conversely, for the middle and upper income groups, it shows a positive impact, with coefficients of 0.298 (significant at the 5% level) and 0.958 (significant at the 1% level), respectively. This indicates significant variations in how monetary policy affects different income groups. In terms of household wealth, the impact of M2 is equally significant. The coefficients of the impact on total household wealth (HW_Total) for the bottom 20% and the middle 30% are 1.174 (1% significant) and 1.766 (1% significant), respectively, suggesting that an increase in the money supply positively contributes to the accumulation of wealth among lower-income households, while higher-income households similarly benefit from the expansion of monetary policy.

**Table 5:** Robustness test: alternative sample.

	Bottom 50%		Middle 40%	Top 10%	Bottom 50%		Middle 40%	Top 10%
	Bottom 20%	Next 30%			Bottom 20%	Next 30%		
	income	income	income	income	wealth	wealth	wealth	wealth
M2	−0.715***	0.298***	0.958***	0.653***	0.439**	0.256***	1.174***	1.766***
	(0.194)	(0.046)	(0.037)	(0.118)	(0.201)	(0.044)	(0.042)	(0.101)
Edu	0.040	0.053**	0.051***	0.004	0.076	−0.013	0.044*	0.002
	(0.076)	(0.011)	(0.013)	(0.025)	(0.053)	(0.016)	(0.015)	(0.044)
P_LF	0.129	0.028	0.201***	−0.051	0.125	0.016	0.047	0.104
	(0.206)	(0.039)	(0.036)	(0.097)	(0.132)	(0.043)	(0.060)	(0.100)
W_Total	0.066*	0.036***	0.033***	0.026				
	(0.027)	(0.008)	(0.006)	(0.017)				
FamSize	0.040	−0.014	−0.042***	−0.080***	−0.078*	−0.063***	−0.114***	−0.078*
	(0.033)	(0.005)	(0.008)	(0.016)	(0.023)	(0.007)	(0.010)	(0.053)
H_Income					0.107***	0.030***	0.043***	−0.013
					(0.024)	(0.005)	(0.006)	(0.035)
Cons.	11.356***	6.780***	4.591***	6.995***	5.697***	9.531***	4.736***	3.815***
	(0.911)	(0.270)	(0.149)	(0.480)	(0.902)	(0.195)	(0.279)	(0.874)
Individual effect	YES	YES	YES	YES	YES	YES	YES	YES
Time effect	YES	YES	YES	YES	YES	YES	YES	YES
Time trend	YES	YES	YES	YES	YES	YES	YES	YES
N	3053	5618	6057	1467	3030	4532	5844	1643

Note: Clustering robust standard error in parentheses. ***, ** and * indicate significance at 1%, 5% and 10% levels, respectively. N is the number of samples.

Secondly, the variable used to represent monetary policy is substituted with a weighted one-year lending rate, with the data sourced from Chinese macroeconomic time series. The findings indicate that interest rates exert a significant negative impact on both average distribution of income per family member (INC) and total household wealth (HW_Total) at the 1% significance level. Generally, lower interest rates tend to encourage income and wealth growth across all households.

Additionally, the influence of expansionary monetary policy on inequality is analyzed by classifying households. As was shown in Table 6, the results suggest that this policy, characterized by lower interest rates, has a significantly stronger impact on high-income households than on those in the lower-middle class, alongside a more pronounced effect on wealth. This observation aligns with the baseline regression outcomes. Furthermore, the regression findings do not show significant differences from the baseline results when substituting the variables associated with household income and wealth with gross household income (H_Income), net household wealth (NetWorth), and total household wealth (HW_Total), respectively.

**Table 6:** Robustness test: alternative explanatory variables.

	Bottom 50%		Middle 40%	Top10%	Bottom 50%		Middle 40%	Top10%
	Bottom 20%	Next 30%			Bottom 20%	Next 30%		
	income	income	income	income	wealth	wealth	wealth	wealth
lending rate	27.411***	−14.102***	−38.682***	−22.450***	−12.496*	−13.844***	−43.866***	−103.968***
	(7.526)	(1.616)	(1.524)	(2.673)	(6.241)	(1.049)	(1.504)	(5.594)
Edu	0.070	0.032	0.033	0.028	0.088	−0.022	0.023*	0.047
	(0.046)	(0.014)	(0.008)	(0.016)	(0.057)	(0.014)	(0.013)	(0.042)
P_LF	0.090	0.031	0.170	0.032	−0.055	0.043	0.095*	0.030
	(0.171)	(0.042)	(0.027)	(0.067)	(0.111)	(0.042)	(0.035)	(0.101)
W_Total	0.039*	0.019***	0.046***	0.033***				
	(0.024)	(0.005)	(0.004)	(0.014)				
FamSize	0.045	−0.002	−0.040**	−0.100***	−0.048*	−0.051***	−0.109***	−0.097***
	(0.022)	(0.005)	(0.005)	(0.011)	(0.021)	(0.005)	(0.007)	(0.029)
H_Income					0.076***	0.027***	0.032***	0.013
					(0.013)	(0.004)	(0.005)	(0.025)
Cons.	4.693***	9.588***	8.699***	10.663***	11.602***	8.737***	13.974***	21.155 ***
	(0.573)	(0.121)	(0.100)	(0.336)	(0.317)	(0.091)	(0.125)	(0.435)
Individual effect	YES	Yes	YES	YES	YES	YES	YES	YES
Time effect	YES	Yes	YES	YES	YES	YES	YES	YES
Time trend	YES	Yes	YES	YES	YES	YES	YES	YES
N	4140	7011	9193	2237	4656	6854	12716	2400

Note: Clustering robust standard error in parentheses. ***, ** and * indicate significance at 1%, 5% and 10% levels, respectively. N is the number of samples.

Ultimately, we conducted panel regressions using the Tobit model to more effectively tackle the issues associated with negative values and missing data for the explanatory variables, particularly where per capita income is set to zero. The results from the Tobit model were compared with those obtained from the previously used ordinary least squares. Furthermore, the regression analysis for households across various income levels—specifically focusing on the lower, middle, and upper tiers—revealed notable differences among these groups. Importantly, the impact of expansionary monetary policy was found to be more pronounced for high-income and high-wealth households, thereby reinforcing the robustness of the findings.

### Analysis of influence mechanism

Our baseline model indicates that expansionary monetary policy significantly influences household income and wealth inequality, with a more pronounced effect on wealth inequality. To gain deeper insights into the economic factors affecting household wealth, we performed a detailed analysis to examine the impact of specific monetary policies on each component of household wealth. The data suggests that an increase in M2 has a notable negative effect on land assets (−0.805, 1% significance), which may indicate that the supply and demand dynamics in the land market are disrupted by monetary policy actions, leading to a decline in land value. On the contrary, the effect of M2 on housing value (H_Value) is positive with a coefficient of 1.336 (1% significance), indicating that expansionary monetary policy effectively promotes the prosperity of the real estate market. In addition, the significant positive impact on financial assets (2.359, 1% significance) suggests that the growth in money supply facilitated households’ investment and wealth accumulation in the financial market.

Table 7 showed the impact of monetary policy on each component of household wealth. In terms of the impact of education, labor, and total income factors, the level of education (Edu) shows a significant positive push on both land and housing values (H_Value), especially on housing values with a coefficient of 0.354 (1% significance). The effect of labor income (P_LF) on durable goods assets is equally significant (0.221, 1% significance), reflecting the optimizing effect of household economic participation on wealth structure. However, the negative effect of family size (FamSize) on financial assets (−0.049, 1% significance) implies that the increase in household members may have diluted the concentration of household wealth to some extent, affecting the ability of households to invest financially. This is consistent with the findings of Liu and Tang (2010) on the rising property income inequality in China.

**Table 7:** Impact of monetary policy on each component of household wealth.

	land asset	house value	finance asset	fixed asset	durables asset
M2	−0.805***	1.336***	2.359***	−0.626	2.709***
	(0.058)	(0.052)	(0.133)	(0.237)	(0.076)
Edu	−0.102***	0.354***	0.330***	0.384***	0.335***
	(0.016)	(0.011)	(0.021)	(0.023)	(0.016)
P_LF	−0.002	0.021	−0.083	0.086	0.221***
	(0.037)	(0.041)	(0.080)	(0.138)	(0.056)
H_Income	0.268***	0.150***	0.221***	−0.014	0.212***
	(0.008)	(0.007)	(0.018)	(0.015)	(0.008)
FamSize	0.021***	0.00038	−0.049***	0.007	0.102***
	(0.009)	(0.007)	(0.012)	(0.013)	(0.00724)
Cons.	13.615***	0.069	−6.877***	7.831***	−6.427***
	(0.283)	(0.151)	(0.459)	(1.440)	(0.417)
Individual effect	Yes	Yes	Yes	Yes	Yes
Time effect	Yes	Yes	Yes	Yes	Yes
Time trend	Yes	Yes	Yes	Yes	Yes
N	16917	30859	20797	6887	24068

Note: Clustering robust standard error in parentheses. ***, ** and * indicate significance at 1%, 5% and 10% levels, respectively. N is the number of samples.

Additionally, we identify a significant link between total household wealth (HW_Total) and property values (H_Value). Therefore, we will consider property value as a dependent variable in our primary model and incorporate an interaction term related to money supply (M2) to explore how the increase in housing prices, driven by expansionary monetary policy, contributes to heightened household wealth inequality. The model can be expressed as follows:


$$NetWorth_{i,t}=\beta_{0}+\beta_{1}MP_{t}+\beta_{2}M2*H\_Value+\beta_{3}H\_Value+\beta_{cv}CV_{i,t}+NF_{i}+YF_{t}+\xi_{i,t}$$ (3)

Table 8 demonstrates a comparison of the impact of major assets on total household wealth (HW_Total), and the data indicate a significant positive correlation between M2 (broad money supply) and household wealth. Specifically, the coefficient of the impact of M2 on total household wealth is 0.678 and is significant at the 1% significance level, indicating that an increase in M2 can effectively enhance household wealth. In addition, the coefficient of housing value (H_Value) on total household wealth is 0.722 (1% significant), further emphasizing the central role of real estate in household wealth. The coefficient of the interaction term between M2 and housing value is 0.260 (1% significant), suggesting that the joint effect of the two plays an important role in the enhancement of household wealth. With regard to financial assets, the impact of financial assets on total household wealth is equally significant, with impact coefficients of 0.105 and 0.056 (both at the 1% significance level), suggesting that a household’s allocation of financial assets has a positive impact on wealth accumulation.

**Table 8:** Comparison of the impact of major assets on household wealth.

	wealth	Wealth	wealth	wealth
M2	0.678***	2.214***	2.250***	0.477***
	(0.024)	(0.043)	(0.061)	(0.054)
H_Value	0.722***			0.663***
	(0.007)			(0.014)
M2*house value	0.260***			
	(0.025)			
finance asset		0.105***		0.056***
		(0.006)		(0.006)
M2*finance asset		0.340***		
		(0.022)		
durables asset			0.119***	0.038***
			(0.006)	(0.005)
M2*durable asset			0.157***	
			(0.030)	
Edu	−0.003	0.031*	0.013	−0.001
	(0.008)	(0.024)	(0.023)	(0.013)
P_LF	0.084**	0.143**	0.144**	0.098**
	(0.023)	(0.038)	(0.045)	(0.037)
H_Income	0.057***	0.062***	0.137***	0.011
	(0.004)	(0.006)	(0.007)	(0.008)
FamSize	−0.260***	−0.253***	−0.178***	−0.194***
	(0.004)	(0.013)	(0.007)	(0.008)
Cons.	0.869***	−0.040	−0.170	0.488***
	(0.168)	(0.204)	(0.291)	(0.187)
Individual effect	YES	YES	YES	YES
Time effect	YES	YES	YES	YES
Time trend	YES	YES	YES	YES
N	0.569	0.266	0.335	0.921

Note: Clustering robust standard error in parentheses. ***, ** and * indicate significance at 1%, 5% and 10% levels, respectively. N is the number of samples.

The impact coefficient of durable goods assets is 0.119 (1% significance), while the interaction term between M2 and durable goods assets is 0.157 (1% significance), showing the value-added effect of durable goods in total household wealth (HW_Total). The impact coefficient of education level (Edu) is close to zero, suggesting a relatively limited role of education factors in wealth accumulation. The effects of labor income (P_LF) and total income (H_Income) are 0.084 and 0.057 (both at the 1% significance level), respectively, indicating that labor market participation and income level contribute significantly to household wealth. The effect of family size (FamSize) on wealth is negative, with a coefficient of −0.260 (at 1% significance), suggesting that an increase in the number of household members may inhibit wealth accumulation.

The effect of expansionary monetary policy on the increase in housing prices in China operates through various channels. The analysis indicates a strong positive relationship between the residential sales price index and M2 since 2005, with regression results achieving significance at the 1% level. This suggests that a rise in M2 significantly contributes to higher housing prices (H_Value). These results align with the work of Domanski et al., Zhou and Wang, and Mumtaz and Theophilopoulou [51–53], further confirming the impact of expansionary monetary policy on China’s real estate market.

Subsequent analysis utilizing the Local Projections method demonstrates the enduring influence of monetary policy shocks on housing prices (H_Value). It indicates that favorable monetary policy (M2) can significantly elevate housing prices, with effects lasting nearly two years. This implies that such policies may affect short-term housing prices while also having lasting implications on total household wealth (HW_Total) dynamics, potentially exacerbating wealth inequality. Therefore, the connection between expansionary monetary policy and housing prices in China is strong, underscoring its role in contributing to the increase in household wealth disparity..

### Discussion on the impact of real estate on urban-rural and regional development differences

As China’s economy continues to grow, the disparities between urban and rural areas are becoming increasingly clear, particularly regarding average distribution of income per family member (INC) and total household wealth (HW_Total) accumulation. The real estate market (H_Value) plays a crucial role in fostering economic growth and significantly affects the financial conditions of households in both urban and rural settings. Nevertheless, notable differences exist between these areas in terms of economic progress, resource distribution, and policy assistance, which all contribute to the widening gap in urban-rural development inequality.

The findings in Table 9 highlight important differences in how the real estate market influences household income and wealth in urban compared to rural areas. Regarding income, the M2 coefficient in urban regions is 1.652, which is significant at the 1% level, demonstrating a considerable positive effect of monetary expansion on urban household income (INC). In contrast, in rural areas, the coefficient of M2 is 0.900. While this remains significant, the overall impact is notably less than that observed in urban settings, illustrating the relatively weaker transmission of monetary policy effects in rural regions.

**Table 9:** Regression results grouped by urban and rural area.

	(1) Urban	(2) Rural	(3) Urban	(4) Rural
	income	Income	wealth	wealth
M2	1.652***	0.900***	0.536***	0.240***
	(0.082)	(0.080)	(0.050)	(0.043)
H_Value			0.862***	0.577***
			(0.016)	(0.012)
finance asset			0.068***	0.089***
			(0.004)	(0.004)
durables asset			0.045***	0.078***
			(0.003)	(0.007)
Edu	0.163***	0.211***	−0.001	−0.028
	(0.031)	(0.031)	(0.013)	(0.012)
P_LF	0.280***	0.480***	0.077**	0.039
	(0.052)	(0.086)	(0.044)	(0.058)
FamSize	−0.041**	−0.040**	−0.207***	−0.206***
	(0.013)	(0.008)	(0.009)	(0.009)
W_Total	0.067***	0.197***		
	(0.014)	(0.009)		
H_Income			0.008	0.057***
			(0.010)	(0.007)
Cons.	−1.260***	−0.846**	0.587**	2.249***
	(0.254)	(0.370)	(0.205)	(0.263)
Individual effect	YES	YES	YES	YES
Time effect	YES	YES	YES	YES
Time trend	YES	YES	YES	YES
N	11928	12135	8037	7882

Note: Clustering robust standard error in parentheses. ***, ** and * indicate significance at 1%, 5% and 10% levels, respectively. N is the number of samples.

In terms of total household wealth (HW_Total), the coefficient on the impact of housing value (H_Value) for urban households, at 0.862, is equally significant, showing the wealth-enhancing effect of growth in the real estate market. In contrast, the coefficient of housing value in rural areas is 0.577, which is also significant, but the lower value reflects the greater challenges rural households face in accumulating wealth. In addition, rural households rely more on durable assets, as evidenced by a higher coefficient of influence on durable goods assets (0.078) than in urban areas (0.045).

Studies have shown that China’s real estate market exhibits significant regional differences, especially in the eastern region where housing prices (H_Value) are significantly higher than in the central and western regions (Meng et al., 2017). In addition, the transmission characteristics of expansionary monetary policy (M2) in different regions also show obvious heterogeneity. The effects of expansionary monetary policy differ significantly between developed and less developed regions due to differences in the level of regional economic development (Shen et al., 2011). Households in economically developed regions are usually able to benefit from monetary policy more quickly, driving income (INC) and wealth growth, while households in less developed regions face larger lagged effects.

The regression outcomes presented in Table 10 indicate notable variations in how the real estate market (H_Value) affects household income (INC) and total household wealth (HW_Total) across different regions, further highlighting the disparities in China’s regional development. In more developed areas, the coefficient of M2 is 2.091, significant at the 1% level, which suggests that the positive influence of monetary expansion policies on households is substantial. Conversely, in less developed regions, the coefficient of M2 stands at 1.924, reflecting a comparatively lower impact and indicating that the effects of monetary policy vary across regions, particularly in terms of income growth, with developed areas experiencing greater benefits.

**Table 10:** Regression results in different regions.

	(1) Developed	(2) less-developed	(3) Developed	(4) Less-Developed
	income	Income	wealth	wealth
M2	2.091***	1.924***	0.388***	0.338***
	(0.101)	(0.083)	(0.110)	(0.077)
H_Value			0.722***	0.454***
			(0.026)	(0.012)
finance asset			0.053***	0.053***
			(0.006)	(0.005)
durables asset			0.021***	0.077***
			(0.005)	(0.006)
Edu	0.151***	0.127***	0.013	−0.021
	(0.036)	(0.035)	(0.023)	(0.018)
P_LF	0.556***	0.695***	−0.025	0.013
	(0.124)	(0.078)	(0.053)	(0.050)
FamSize	−0.094***	−0.043**	−0.276***	−0.232***
	(0.017)	(0.015)	(0.015)	(0.014)
W_Total	0.079***	0.159***		
	(0.014)	(0.018)		
H_Income			0.007	0.032**
			(0.017)	(0.012)
Cons.	−2.066***	−1.722***	0.002	1.754***
	(0.413)	(0.298)	(0.426)	(0.321)
Individual effect	YES	YES	YES	YES
Time effect	YES	YES	YES	YES
Time trend	YES	YES	YES	YES
N	3305	8459	3175	8600

Note: Clustering robust standard error in parentheses. ***, ** and * indicate significance at 1%, 5% and 10% levels, respectively. N is the number of samples.

In terms of total household wealth, the impact coefficient of housing values in developed regions is 0.722, showing that positive growth in the real estate market contributes directly to the increase in household wealth. In contrast, in the less developed regions, the impact coefficient of housing value is 0.454, which, although also significant, is significantly lower than in the developed regions, reflecting the fact that the less developed regions face greater constraints on wealth growth. In addition, the impact coefficient of durable goods assets is higher in the less developed regions (0.077) than in the developed regions (0.021), which may indicate that households in the less developed regions rely more on consumer durables.

These results suggest that the performance of the real estate market not only exacerbates interregional development gaps but also further deepens social inequality, suggesting that policymakers should pay more attention to interregional differences in the implementation of monetary policy in order to promote more balanced economic development.

### Further analysis: is financial literacy another factor?

Financial literacy serves as an essential indicator of a household’s financial capability and can greatly influence how well a household manages challenges posed by monetary policy. According to Friedrich et al. [54], families with at least one member who possesses a degree are more likely to lessen and gain from the negative impacts of monetary expansion. This finding underscores the significance of integrating financial literacy with educational qualifications in understanding the economic status of households. In terms of intergenerational effects, the role of financial literacy in promoting income mobility is also confirmed. According to Zhao and Wu [55], financial literacy reduces the impact of parental income on children, which in turn promotes intergenerational income mobility. This suggests that the financial literacy of a family not only affects the current economic situation, but also helps to break the cycle of poverty and enhance economic opportunities for the next generation. Trond-Arne Borgersen studied non-profit third-housing sector (THS) into a housing market with Cournot competition, and the results laid the foundation for housing policy formulation [56].

Table 11 demonstrates the different responses of high and low financial literacy households in response to monetary policy (M2). The results show that high financial literacy households are significantly more sensitive to average distribution of income per family member (INC) and total household wealth (HW_Total) growth than low financial literacy households. Specifically, the responses of high financial literacy households to M2 are 1.573 (income) and 2.998 (wealth), while the corresponding values for low financial literacy households are 1.692 (income) and 2.408 (wealth). This suggests that households with higher financial literacy are able to more effectively utilize the opportunities presented by monetary policy for income and wealth growth.

**Table 11:** Regression Results in Different Levels of Financial Literacy.

	(1) High	(2) Low	(3)High	(4)Low
	income	Income	wealth	wealth
M2	1.573***	1.692***	2.998***	2.408***
	(0.118)	(0.042)	(0.171)	(0.044)
Edu	0.127**	0.134	0.002	0.040**
	(0.040)	(0.024)	(0.052)	(0.015)
P_LF	0.408**	0.623***	0.198	0.185**
	(0.088)	(0.064)	(0.150)	(0.037)
FamSize	−0.104**	−0.040***	−0.113*	−0.173***
	(0.026)	(0.010)	(0.040)	(0.010)
W_Total	0.071**	0.129***		
	(0.025)	(0.013)		
H_Income			0.115*	0.111***
			(0.047)	(0.010)
Cons.	−1.108*	−0.804***	−3.129***	−0.642**
	(0.474)	(0.211)	(0.728)	(0.205)
Individual effect	YES	YES	YES	YES
Time effect	YES	YES	YES	YES
Time trend	YES	YES	YES	YES
N	3297	26415	3389	27594

Note: Clustering robust standard error in parentheses. ***, ** and * indicate significance at 1%, 5% and 10% levels, respectively. N is the number of samples.

In addition, the effect of education level (Edu) also differs significantly across financial literacy households. The impact of education level on income is 0.127 for high financial literacy households compared to 0.134 for low financial literacy households, which is still a positive effect despite the smaller contribution of education in low financial literacy households. Furthermore, the impact of labor income (P_LF) is also higher in high financial literacy households (0.408) than in low financial literacy households (0.623), suggesting a closer relationship between labor market participation and income growth in high financial literacy households, further emphasizing the critical role of financial literacy in household financial capability.

## Conclusions and implications

This research systematically examines the effects of expansionary monetary policy on household income and wealth, utilizing CFPS 2010–2020 microdata to explore its mechanisms. The findings indicate that expansionary monetary policy has played a role in enhancing household income and wealth overall; however, its effects vary significantly among different types of households, contributing to the widening of income and wealth disparities.

Furthermore, the influence of monetary policy on household wealth was notably greater than its impact on income, primarily operating through the real estate market mechanism. The rise in property values following the implementation of this policy, particularly in urban and metropolitan areas, significantly boosted the wealth levels of the population. Nonetheless, this wealth increase has not benefited all households equally; rather, it has exacerbated the wealth gap between urban and rural areas, highlighting the limitations of current policies in promoting equity.

In the policy debate, governments should clarify whether their support measures are targeted at the supply or demand side of the market. While social housing programs can provide a measure of housing security for low-income households, a potential side effect is that they may raise the price of commercial housing and squeeze out groups that are not included in social housing programs. Therefore, policymakers need to carefully assess the relationship between social housing and market housing to avoid further exacerbating market inequality.

Based on these findings, this study recommends that policymakers adopt a broader policy mix, focusing on how monetary policy interacts with other public funding mechanisms to more fully influence economic outcomes. In particular, the government should consider the synergies between different types of public investment and draw on successful experiences in other areas to promote sustainable economic development. At the same time, it should continue to adhere to the real estate market control policy of “housing without speculation” to ensure the stability and healthy development of the market. In addition, a long-term redistribution mechanism should be established, and consideration should be given to introducing inheritance tax, property tax and capital gains tax to mitigate the impact of wealth concentration on social inequality. Enhancing the financial literacy and investment capacity of low-income households is an important way to achieve shared prosperity. This will not only help alleviate social inequality caused by widening income and wealth gaps, but will also promote overall social progress. Future research should further explore the interaction between monetary policy and wealth distribution in different economies in order to attract more international academic attention and discussion.

## Supporting information

S1 Data (A) cfps1 famecon 1(B); cfps famecon 2; (C)China M2M1M0 Money Supply. (RAR)
